# Heat exposure modulates redox balance and gonadal-related hormones in Wistar rats

**DOI:** 10.5935/1518-0557.20250046

**Published:** 2025

**Authors:** Damilare Emmanuel Rotimi, Akinbowale Ewaoluwa Shalom, Oluyomi Stephen Adeyemi

**Affiliations:** 1 Department of Biochemistry, Landmark University, Omu-Aran 251101, Nigeria; 2 Department of Biochemistry, Medicinal Biochemistry & Toxicology Laboratory, Bowen University, Iwo 232101, Nigeria

**Keywords:** climate change, global warming, reproductive toxicology, thermal stress

## Abstract

**Objective:**

This study assessed the effect of heat exposure on gonadal-related hormones in male and female Wistar rats.

**Methods:**

Twenty rats (140 - 160) were divided into 2 groups (male and female rats) and further sub-grouped into control and heat-exposed rats. Rats were exposed to heat for 4 hours daily for 21 days after which the rats were necropsied, and blood collected from the jugular veins.

**Results:**

Heat exposure caused appreciable decreases in the levels of luteinizing hormones and follicle-stimulating hormones in both the male and female rats., Also, heat exposure caused a decline in the level of testosterone in male rats and decreased progesterone and oestrogen in their female counterparts. Furthermore, heat exposure caused oxidative stress by elevating the levels of malondialdehyde and nitric oxide, but lowering the antioxidants such as catalase, superoxide dismutase and thiols in rats.

**Conclusions:**

This study thus showed that heat exposure significantly disrupts hormonal balance and induces oxidative stress in both male and female Wistar rats. Findings have implications for climate change impact on health.

## INTRODUCTION

During the last century, the global mean temperature has increased significantly due to climate change, which has also been related to the marked increases in the frequency and intensity of heat waves (extreme heat events). Several negative impacts on human health have been linked to climate change. Dehydration, heat exhaustion and heat stroke are common side effect of climate-related heat exposure, which can result in acute mortality or worsen preexisting chronic conditions (Glaser *et al*., 2016). The response to heat stress is a complex process comprising neuroendocrine regulation, energy metabolism, and immune response (Martelli & Nannoni, 2021). Heat stress results in numerous compensatory changes in body metabolism, behaviour, and physiological functions (Valente *et al*., 2013), increased skin and body core temperatures, enzymatic reactions, levels of circulating cortisol and corticosterone and changes in endocrine and reproductive processes (Li *et al*., 2016).

Heat stress decreases spermatozoa production and motility (Agarwal *et al*., 2012). Additionally, testicular heat stress has been found to inhibit the production of gonadal hormones such as testosterone and estrogen (Jeremy *et al*., 2021) or decrease the circulating levels of testosterone, follicle stimulating hormone (FSH), luteinizing hormone (LH) and estrogen (Diamanti-Kandarakis *et al*., 2009). Similarly, the female gonads were found in several investigations to exhibit some sort of malfunction upon exposure to heat (Valente *et al.*, 2013). In both acute and chronic heat-stressed animals, abnormal granulosa cell phenotypes with oocyte separation from the granular cell layer were observed (Bei *et al*., 2020). Chronic heat stress has been associated with a drop in estradiol concentrations, which is consistent with morphological alterations in granulosa cells (An *et al*., 2020) but there are no reports on the plasma estradiol concentrations. This could be explained by altered expression patterns of the enzyme aromatase, the primary hormone in the manufacture of estrogen, in response to heat stress (Li *et al*., 2016). Gonadotropin receptors on the cell surface exhibit decreased expression of the FSH receptor after acute heat stress in rats, as well as increased expression of the FSH receptor in chronic heat-stressed rats, despite the destruction of granulosa cells (Shimizu *et al*., 2005).

Gonadal hormones play an important role in the control of numerous CNS processes, particularly mood and cognitive functions, during fertile life and reproductive age (Van Der Leeuw *et al*., 2013). Due to its effects on both male and female gonadal hormones, heat exposure can have a major influence on the reproductive system. Stress from heat can change the expression of reproductive hormones, which may have an impact on fertility (Sukhan *et al.,* 2021). To identify and reduce potential health hazards, it is essential to understand how thermal heat affects the hormonal systems. Thus, this study assessed the effect of heat exposure on the gonadal hormones in male and female rats.

## ALS AND METHODS

### Experimental Animals

The rats were acquired from the animal housing facility at the Department of Biochemistry, University of Ilorin in Nigeria. They were housed in clean and well-ventilated cages, ensuring their hygiene and comfort. Additionally, they were given unlimited access to both food and water, and they had seven days to acclimate before treatment started. The animals were kept in a 24**±**3**˚C** laboratory environment with 12 hours of darkness, 12 hours of light, and a relative humidity of 60**±**5%. The Landmark University Ethics Committee authorized the study protocol (LUAC/BCH/2024/005A).

### Animal Grouping

Twenty (20) healthy Wistar strain rats weighing between 140 and 160 g were used for this experiment. The animals were grouped into different gender of ten (10) male and ten (10) female each. Each of these groups were further subdivided into two (2) different groups each, making it four (4) groups, each having five (5) rats. For the male or female sub-groupings, there were a control (non-heat exposed) and the heat-exposed groups (Two 100 watts bulbs was placed in the cages of the rats being exposed to heat) (Chinko & Umeh, 2023), which was turned on at (39±2^o^C) for 4 hours daily for 21 days (Liao *et al*., 2022). After an overnight fast, the animals were sacrificed using diethyl ether anesthesia. Testes and ovaries were harvested, homogenized with 0.25 M sucrose, and centrifuged. Blood was also collected, centrifuged, and plasma was obtained for biochemical analysis.

### Biochemical Assays

The total protein concentration was carried out based on the principle of cupric ion development with a peptide bond as described by Gornall *et al*. (1949). Superoxide dismutase activity was determined using the technique described by Misra & Fridovich (1972). To measure the catalase activity was based on the ability of catalase to convert hydrogen peroxide (H_2_O_2_) into oxygen and water as described by Beers & Sizer (1952). The procedure described by Beutler *et al*. (1963) was used to evaluate the level of reduced glutathione (GSH). Thiobarbituric acid reactive substances (TBARSs) were used as an indication in Satoh’s (1987) method to measure lipid peroxidation and provide a rough estimate of malondialdehyde (MDA). The method described by Adeyemi *et al*. (2018) was used to determine the nitric oxide level.

Serum hormone concentrations were determined using various immunoassay kits, following Tietz’s protocols (Tietz, 1995). Testosterone was measured with an EIA kit, FSH and LH with an ELISA kit (ElabScience, Houston, Texas 77079, USA), prolactin and progesterone with a microplate immunoenzymometric assay, and GnRH with a competitive ELISA kit.

### Statistical analysis

One-way ANOVA was used to analyse the data. The Dunnett post-hoc test was used for group comparison. The results were expressed as an average of five repetitions±standard error of the mean (SEM). A *p*-value of 0.05 was considered significant. The graphing and analysis were performed on GraphPad Prism (version 9, GraphPad Software Inc., La Jolla, CA).

## RESULTS

### Ratio of the testicular and ovary to rat body weight

Following a period of 21 days of heat exposure, the weights and organs of both male and female groups are shown in Table 1. Heat exposure significantly decreased the relative testicular weight in male rats compared to the control group. No significant changes in animal weight and relative ovary weight in heat-exposed female rats compared to controls.

**Table 1:** Body and organ weight after 21 days of heat exposure in male rats.

	Male		Female	
	Control	Heat-exposed	Control	Heat-exposed
Final weight	212.4±13.42	165±19.67^***^	182.8±9.25	169.5±5.01
Initial weight (g)	146.2±8.38	154.2±7.50	146.6±9.21	155±6.32
Body weight change (%)	45.28±3.5	7.46±1.2	24.46±2.6	9.35±1.4
Testicular weight	2.57±0.18	2.31±0.17	0.40±0.09	0.35±0.16
Relative testis weight (%)	0.64±0.2	1.39±0.17^****^	0.62±0.1	0.73±0.1

Each value is a mean of five replicates±SD. Data with asterisks are significantly different at

*** at *p*<0.001 and

**** at *p*<0.0001.

### Gonadal total protein concentration

**Figure 1:**
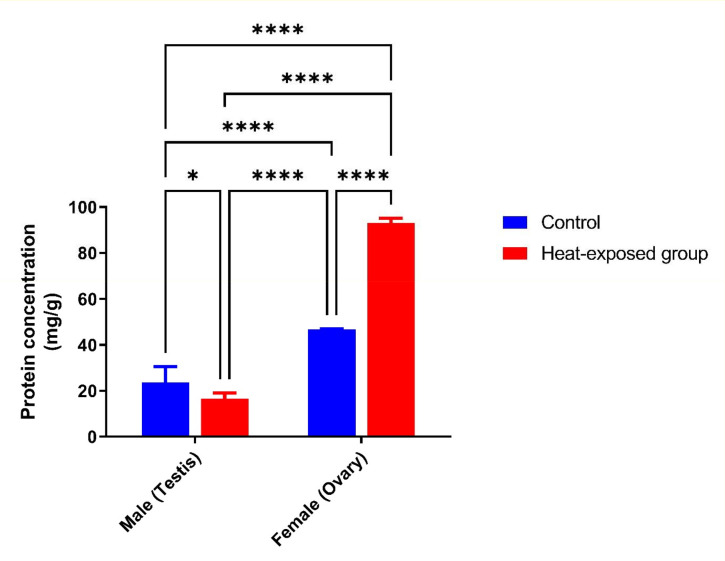
Effect of heat exposure on rat gonad protein concentration in male and female rats after 21 days of exposure.

The concentration of gonadal protein was lower in males compared to females. Notably, exposure to heat led to a decrease in the concentration of protein in rat male gonads. In contrast, heat exposure led to a higher concentration of proteins in the gonads of females (Figure 1).

Each bar is a mean of five replicates±SD. Data with asterisks are significantly different at *p*<0.05, ** at *p*<0.01, *** at *p*<0.001 and **** at *p*<0.0001.

### Gonadal oxidative stress markers

**Figure 2:**
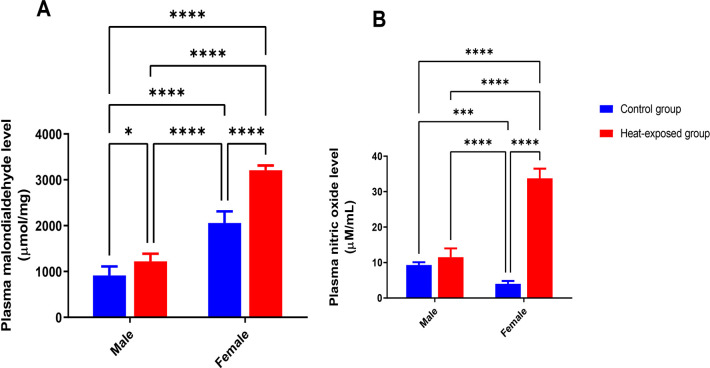
Effect of heat exposure on oxidative stress markers in the gonads of male and female rats. [A] Malondialdehyde (MDA) level; [B] Nitric oxide (NO) level.

Figure 2A shows the malondialdehyde (MDA) levels in the gonad of male and female rats. The MDA level in the gonads increased significantly in the heat-exposed group compared to that of the control group. After 21 days of heat exposure, there was a significant increase in MDA level in heat-exposed male rats compared to the male control group. Similarly, there was also a significant increase in the MDA level of the female rats exposed to heat compared with the female control group.

Each bar is a mean of five replicates±SD. Data with asterisks are significantly different at *p*<0.05, ** at *p*<0.01, *** at *p*<0.001 and **** at *p*<0.0001.

Figure 2B displays the nitric oxide (NO) level in the gonads of male and female rats. The male rats exposed to heat had elevated level of NO compared with the control male group. In the heat-exposed female rats, there was a significant increase in NO level compared with the control female group.

### Gonadal antioxidant activity

**Figure 3:**
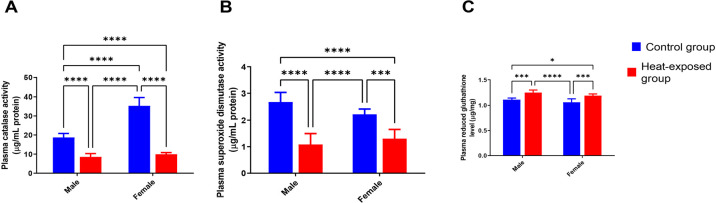
Effect of heat exposure on [A] catalase activity (CAT); [B] superoxide dismutase activity (SOD); [C] reduced glutathione (GSH) level in the gonads of male and female rats.

Figure 3A shows the catalase activity in the gonads of male and female rats. The heat-exposed male rats showed a significant decrease in catalase activity compared with the male control group. The heat-exposed female rats showed a significant decrease in catalase activity compared with the female control group. Meanwhile, in the control male group the catalase activity was lower compared with the control female group.

Each bar is a mean of five replicates±SD. Data with asterisks are significantly different at *p*<0.05, ** at *p*<0.01, *** at *p*<0.001 and **** at *p*<0.0001.

Figure 3B shows the superoxide dismutase (SOD) activity in the gonads of male and female rats. The heat-exposed male rats had lower SOD activity compared with the male control group. In the heat-exposed female group, there was a significant decrease in SOD activity compared with the female control rats.

Figure 3C below shows the reduced glutathione (GSH) level in the gonads of male and female rats. In male rats exposed to heat, GSH level decreased significantly compared with the male control group. In the heat-exposed female group, there was a significant increase in GSH level compared with the female control rats.

### Levels of gonadal-related hormones in rats

**Figure 4:**
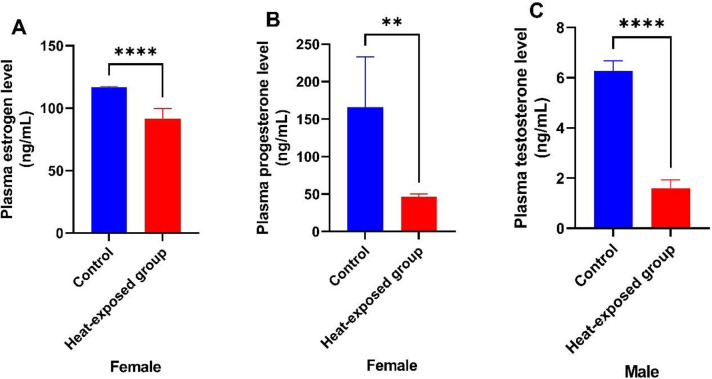
Effect of heat exposure on [A] estrogen level in female rats; [B] progesterone level in female rats; [C] testosterone in male rats.

There was a significant decrease in the estrogen and progesterone level in the heat-exposed female group compared with their control counterparts (Figure 4A and B). Figure 4C shows the testosterone level in male rats. Similarly, heat exposure led to a significant decrease in the testosterone level compared with the control group.

Each bar is a mean of five replicates±SD. Data with asterisks are significantly different at *p*<0.05, ** at *p*<0.01, *** at *p*<0.001 and **** at *p*<0.0001.

**Figure 5:**
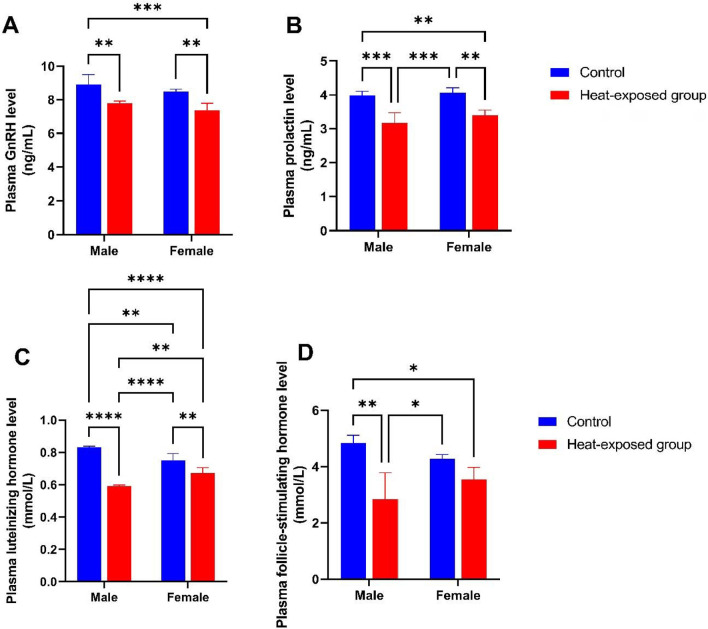
Effect of heat exposure on levels of [A] gonadotrophin-releasing hormone (GnRH); [B] Prolactin; [C] luteinizing hormone (LH); [D] follicle stimulating hormone (FSH) in male and female rats.

Figure 5A shows the level of gonadotrophin-releasing hormone (GnRH) in male and female rats. Heat exposure caused a significant decrease in the GnRH level in both male and female rats compared with their respective control counterparts.

Each bar is a mean of five replicates±SD. Data with asterisks are significantly different at *p*<0.05, ** at *p*<0.01, *** at *p*<0.001 and **** at *p*<0.0001.

Figure 5B shows the prolactin level in male and female rats. Heat exposure led to a significant decrease in the prolactin level in both male and female rats compared with their respective control counterparts.

Figure 5C shows the luteinizing hormone (LH) levels in male and female rats. There was a significant decrease in the LH level of the heat-exposed male rats compared with the male control group. Similarly, there was a significant decrease in the LH level of the heat-exposed female rats compared with the female control group. Furthermore, a significant decrease in the LH level was observed in the male rats exposed to heat compared with their female counterparts.

Figure 5D shows the follicle stimulating hormone (FSH) level in male and female rats. There was a significant decrease in the FSH level of the heat-exposed male group compared with the male control rats. A significant decrease in the FSH level was observed in the heat-exposed female rats compared with the female control group.

## DISCUSSION

Gonadal hormones play crucial roles in reproductive functions and overall health. However, these hormones are sensitive to environmental factors, including temperature. Heat exposure can affect the endocrine system, potentially altering the secretion and function of these hormones (de La Salles *et al*., 2017). Heat stress consequent of climate change and resultant global warming could impair reproductive function in both male and female rats by altering key hormones of the hypothalamic-pituitary-gonadal axis (An *et al*., 2020). Decreased testosterone in males and disrupted estrous cycles in females demonstrate the detrimental effects of heat stress on mammalian reproduction (Lu *et al*., 2021). The present study investigated the comparative effect of heat exposure on the gonadal-related hormones in male and female rats.

The body weight loss alongside the decreases in the weights of reproductive organs in the male and female rats exposed to heat might be due to thermal shock on the animals (Kang & Kim, 2021). This is likely due to heat-induced oxidative stress impairing cell membranes and nucleic acids, leading to apoptosis of Sertoli and germ cells (Ngoula *et al*., 2020).

The protein concentration provides details about the overall health of the animals, as proteins are necessary for the growth and health of the body’s cells and tissues. In the present study, the decrease in the protein concentration of rats exposed to heat raises the possibility that heat stress predisposes to the loss of cellular proteins (Frier & Locke, 2007).

Since malondialdehyde (MDA) is one of the most well-known by-products of lipid peroxidation, its level has long been used as a lipid peroxidation marker in studies on oxidative stress and redox signals (Morales & Munné-Bosch, 2019). The increase in MDA level of the male and female heat-exposed rats suggest lipid peroxidation likely resulting from oxidative stress. Similar results have previously been obtained by Khafaga *et al*. (2019) and Morrell (2020) which showed that heat exposure resulted in oxidative stress in rats. Furthermore, the elevated NO level in rats following heat exposure might contribute to oxidative or nitrosative stress (Varasteh *et al*., 2018). Collectively, heat exposure might promote the generation of free radicals (ROS) (Rahman & Rahman, 2021) leading oxidative stress and resultant cellular damage.

To reinforce that heat exposure might have caused oxidative stress, t study, we observed a concomitant decrease in CAT and SOD levels in rats exposed to heat. The depletion of CAT and SOD levels in heat-exposed rats suggests a reduced free radical scavenging capacity (Singh *et al*., 2021). The results are similar to those of an experiment carried out by Gupta *et al*. (2021). Meanwhile, the GSH level increased following heat exposure. The increase in the GSH level might be an adaptive response in rats to cope with the heat stress-induced oxidative assault (Habashy *et al*., 2019).

The main female sex hormones, estrogens, oversee secondary sexual traits that emerge during puberty and sexual maturity, as well as the regulation of the operations of the female reproductive system (Fuentes & Silveyra, 2019). Progesterone is a pregnancy-related hormone that reduces myometrial contractility to maintain pregnancy and prepares the endometrium for implantation of the fertilized zygote. It prevents rejection of a half-allogeneic fetus by acting as a natural immunosuppressant in high doses. Numerous other related steroid hormones have it as a precursor (Lamb *et al*., 2020). The main sex hormone in men is testosterone, which is generated in the gonads and female ovaries. It oversees the growth of male reproductive tissues, as well as the development of muscles and traits that define men, such as the development of facial hair (Underwood *et al*., 2021). In the present study, heat exposure caused a significant decrease in the levels of estrogen, progesterone and testosterone compared with the control group. Low levels of gonadal hormones might predispose to various conditions, including Klinefelter syndrome, hypogonadotropic hypogonadism, and infertility (Berdyugina *et al*., 2018).

Gonadotrophin (GnRH) is synthesized in hypothalamic GnRH neurons and secreted into the hypophyseal portal circulation. The GnRH release pattern regulates the synthesis and release of the pituitary gonadotropins, FSH, and LH. GnRH is a decapeptide (Garg & Berga, 2020). The hormone prolactin is a polypeptide that is involved in lactation, breast development, and a host of other homeostasis-maintaining processes. Prolactin shares structural similarities with growth hormone and placental lactogen hormone (Stratakis, 2021) Luteinizing hormone (LH) is a gonadotropic hormone secreted by the pituitary gland. By activating the G-protein coupled LH/choriogonadotropin receptor (LHCGR), which is found on the plasma membranes of Leydig cells, it increases testosterone (T) production by testicular Leydig cells (Huhtaniemi, 2018). Human gonadotropin follicle stimulating hormone (hFSH), as a glycoprotein, plays a fundamental role in the management of fertility in mammalian reproduction and is secreted by the anterior pituitary gland (Rastegari *et al*., 2023). In this study, heat exposure led to appreciable depletion in the level of GnRH and prolactin; this may suppress and alter reproductive function and lactation capacity (Zhang *et al*., 2020; Moustafa, 2021). Furthermore, heat exposure caused a decline in the levels of LH and FSH compared with the control group. The decrease in LH and FSH could reduce the richness of testicular interstitial tissue, which further affects the secretion and synthesis of testosterone, and the reproductive function of the body will be seriously damaged (Lu *et al*., 2021). Reduced levels of LH and FSH can affect the ovulation process in women, potentially leading to infertility or difficulties in conceiving, and **i**n men, low levels of LH and FSH can suggest hypogonadism, a condition in which the testes produce insufficient amounts of sex hormones, including testosterone.

The estrous cycle of female rats was not controlled in this study, as hormonal fluctuations across different cycle stages could introduce variability in baseline hormone levels, potentially influencing the observed effects of heat exposure. We acknowledge this as a limitation and suggest that future studies incorporate estrous cycle monitoring to minimize this variability. Additionally, while our sample size was limited to 20 animals, which may affect the generalizability of the findings, the observed trends provide valuable preliminary insights. Future research with larger, more diverse samples will be essential to validate these findings and further elucidate the molecular mechanisms underlying these effects.

## CONCLUSIONS

Heat exposure adversely caused oxidative stress and lowered gonadal hormones, in manners that might predispose to reproductive inadequacy in both male and female rats. In addition, the present findings underscore the effect of climate change on health. Collectively, our study shows how elevated temperatures could disrupt the hormonal balance and modulate redox balance to affect reproductive health and in the long-term with potential consequences for mammalian fertility.
